# Clinical Insights on My Acute Ischemic Stroke Caused by a Carotid Web

**DOI:** 10.7759/cureus.76093

**Published:** 2024-12-20

**Authors:** Koji Yokoyama

**Affiliations:** 1 Department of Pediatrics, Japanese Red Cross Wakayama Medical Center, Wakayama, JPN

**Keywords:** acute ischaemic stroke, carotid web, cervical ultrasonography, computed tomography imaging, ct imaging, magnetic reasoning imaging (mri)

## Abstract

Acute ischemic stroke, a medical emergency caused by reduced cerebral blood flow, results in brain cell damage. While commonly associated with older individuals, strokes can also occur in young and middle-aged adults, posing significant socio-economic and health challenges due to the long-term impact of the condition. This poses significant socio-economic and health challenges because stroke is a leading cause of disability and mortality. A carotid web (CaW), a rare form of fibromuscular dysplasia, is increasingly recognized as a cause of cryptogenic stroke in young adults. Characterized by a fibrous intimal flap in the internal carotid artery, a CaW disrupts blood flow, promoting thrombosis and increasing stroke risk.

I am a 46-year-old pediatrician with no significant medical history who experienced an ischemic stroke due to a CaW. The onset occurred during a morning jog when I experienced nausea, malaise, and unsteady movement. Although I exhibited no headache or paralysis, my wife observed slurred speech, prompting emergency consultation three hours after onset. Imaging revealed ischemia in the left middle cerebral artery territory. I underwent thrombolysis and mechanical thrombectomy, with good recovery except for mild aphasia. Pathological examination confirmed a CaW in the left carotid artery, with myxoid intimal changes and fibroblast proliferation. While the initial ultrasound (US) failed to detect a CaW, subsequent detailed US identified the lesion. Routine magnetic resonance angiography (MRA) also missed the CaW, highlighting its limitations in detecting such abnormalities.

Diagnostic modalities like digital subtraction angiography (DSA) and computed tomography angiography (CTA) are effective but invasive. Non-invasive alternatives, such as US and MRA, are limited by operator dependency and technical constraints. For instance, B-mode US in experienced hands can detect CaWs in longitudinal views, but its diagnostic accuracy is lower than that of CTA and DSA. Advanced imaging, such as contrast-enhanced US and 3D MRA, offers potential improvements but is not yet standard practice. In my case, CTA provided a definitive diagnosis, while initial US and MRA missed smaller lesions.

My recovery included intensive rehabilitation to address residual aphasia. Although I resumed professional duties within three months, minor symptoms, such as mild numbness and occasional speech difficulty, persist. This case highlights the diagnostic challenges of CaWs, particularly pre-stroke. Further research is needed to improve non-invasive detection methods and understand the pathogenesis of CaWs in order to facilitate earlier diagnosis and prevention.

## Introduction

Acute ischemic stroke is a medical emergency caused by decreased blood flow to the brain, which results in damage to brain cells [1]. While strokes are often associated with older adults, approximately 16% occur in individuals aged 15-49. These strokes, known as cryptogenic strokes, when no clear cause is identified, pose unique challenges in diagnosis and prevention [2,3]. This is particularly concerning given the enormous socio-economic burden of stroke, which is the leading cause of disability, and the significantly elevated risk of death faced by younger stroke patients compared with the general population. Recent studies further highlight the rising incidence of stroke among young adults, emphasizing the urgency of addressing this growing public health issue [4]. A carotid web (CaW) is an unusual form of fibromuscular dysplasia, featuring a fibrous, shelf-like intimal flap from the posterior wall of the internal carotid bulb that projects into the arterial lumen. A CaW disrupts normal blood flow, creating stasis that promotes thrombogenesis and elevates the risk of acute ischemic stroke [5]. I am a 46-year-old in excellent health, as consistently validated through regular medical check-ups, with over 20 years of experience as a general physician, primarily specializing in pediatric care in acute care hospitals, as well as contributing to both basic and clinical research. Here, I will describe the onset of my acute ischemic stroke due to the rare and individual-specific cause of a CaW. This report describes my personal experience with a stroke caused by a carotid web, detailing the diagnostic process, imaging challenges, and treatment outcomes. It aims to shed light on this rare condition and the importance of improving diagnostic strategies to prevent similar events.

## Case presentation

At 5:30 a.m., I began my usual daily jog. Within minutes, I experienced mild nausea, a general sense of malaise, and an inability to run straight, which caused me to repeatedly veer to the left. Despite these symptoms, I returned home, hoping rest would alleviate them. Until that day, I had not experienced nausea or fatigue at all. I soon noticed that I could not run straight; unconsciously, I would run a few steps to the left, stop, and then repeat the pattern many times. Normally, I jog for about 60 minutes, but I decided to cut my jog short after around 10 minutes and returned home. Hoping that a short rest would alleviate my symptoms, I lay down in bed. My family and I had planned to spend the day applying wax to the wooden deck in our courtyard. Although the nausea and malaise persisted, I had breakfast. My wife commented on my pallor, and I realized that I was slurring my speech (Video 1).

**Video 1:** A video of myself immediately after the onset of an acute ischemic stroke I could clearly picture the exact place names in my mind, but when I tried to speak, I could not articulate the correct words; either the endings of the words were incorrect, or I could not say the words at all. I was aware that I was not able to verbalize my thoughts accurately.

**Figure 1:**
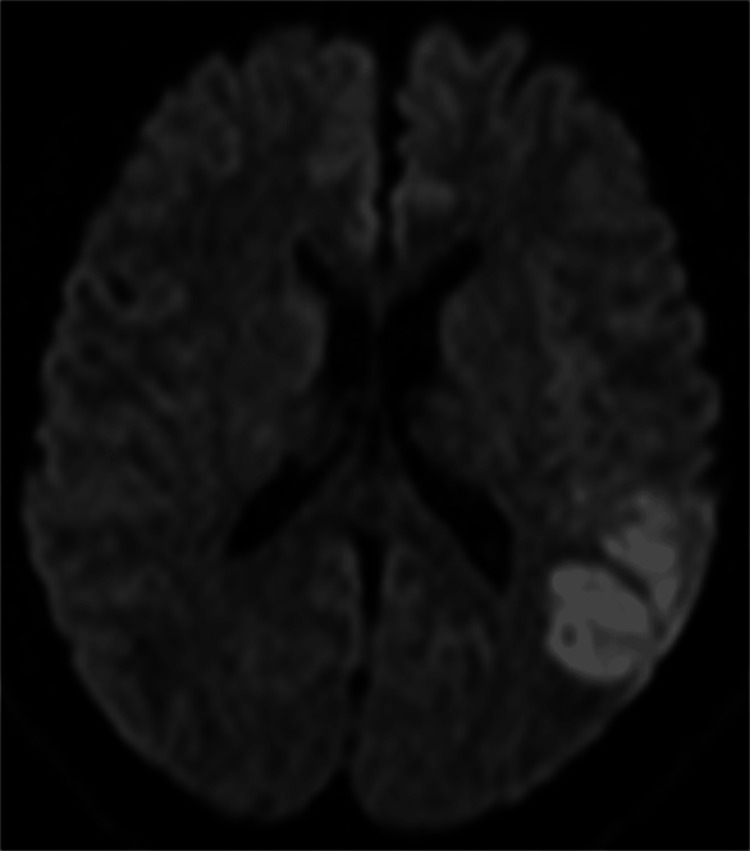
MRI examination of my brain performed on the fourth hour after onset The left temporal lobe, parietal lobe, and insular cortex infarction were revealed by diffusion-weighted MRI.

At the time of presentation, I had no headache, limb paralysis, or sensory deficits. However, slurred speech and occasional incoherence were noted. Although I initially felt only mild discomfort, my wife observed slurred speech and pallor, prompting urgent medical evaluation. My awareness of speech difficulties, including trouble finding the right words, indicated a potential neurological issue. I, a 46-year-old male weighing 64 kg (body mass index 21.4 kg/m^2^), was brought to the emergency department approximately three hours after I had woken up. For the past five years, I have engaged in daily jogging and have participated in local marathon events. I do not consume alcohol or smoke. I have no significant medical history, family history of serious illnesses, or previous hospitalizations. I also consistently undergo occupational health checkups and annual comprehensive health screenings, none of which have revealed any abnormalities. I am independently ambulatory, exhibit no signs of paralysis, and can verbally communicate. However, there are instances where my conversational coherence fluctuates, leading to periods of clear communication interspersed with moments of disorganization. Furthermore, I occasionally give repeated responses. On physical examination, no neurological symptoms were observed, including paralysis or sensory disturbances. My blood pressure was 129/88 mmHg, my pulse was 60 beats/minute, my respiratory rate was 19 breaths/minute, my tympanic temperature was 35.6°C, and my oxygen saturation was 100% on room air. My National Institutes of Health Stroke Scale (NIHSS) score was three out of a maximum of 42 points, comprising a score of two for consciousness and one for best language. A contrast-enhanced computed tomography (CT) scan of my head and neck revealed ischemia in the left middle cerebral artery territory, correlating with my language and comprehension difficulties. Diffusion-weighted magnetic resonance imaging (MRI) showed infarction in the left temporal, parietal, and insular regions, consistent with my aphasia (Figure 1).

**Figure 2:**
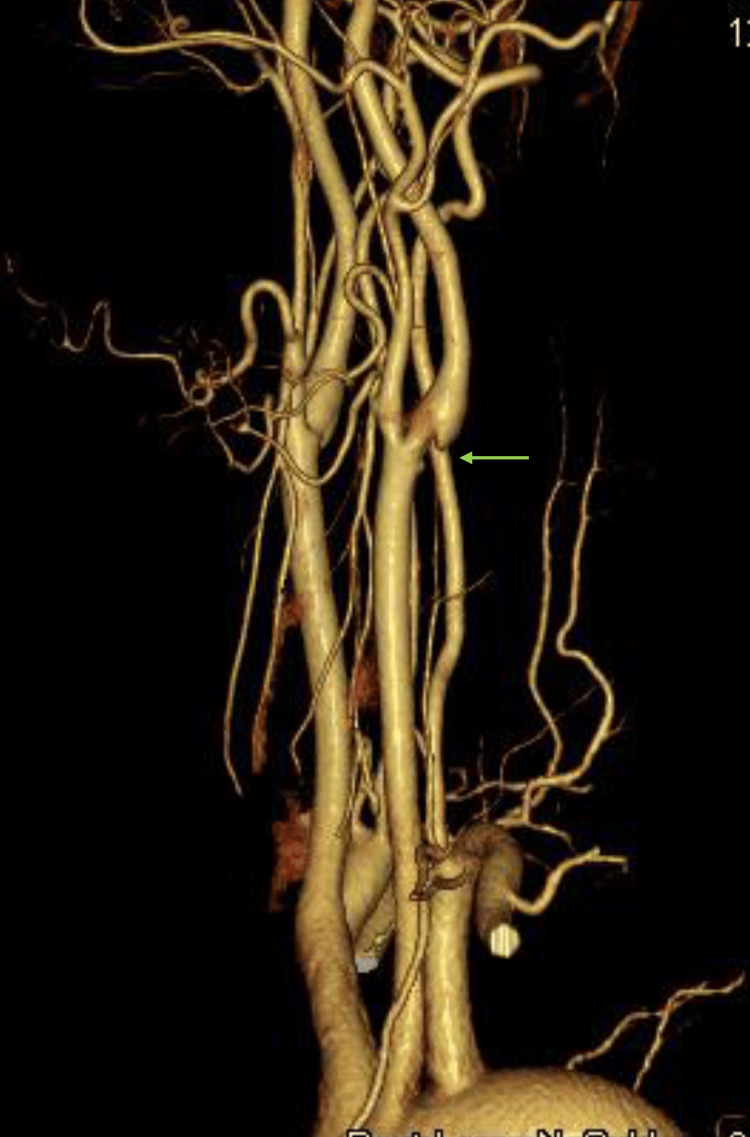
Contrast-enhanced CT scan of the neck at the time of hospitalization A carotid web is observed at the origin of the left internal carotid artery (indicated by the green arrow).

**Figure 3:**
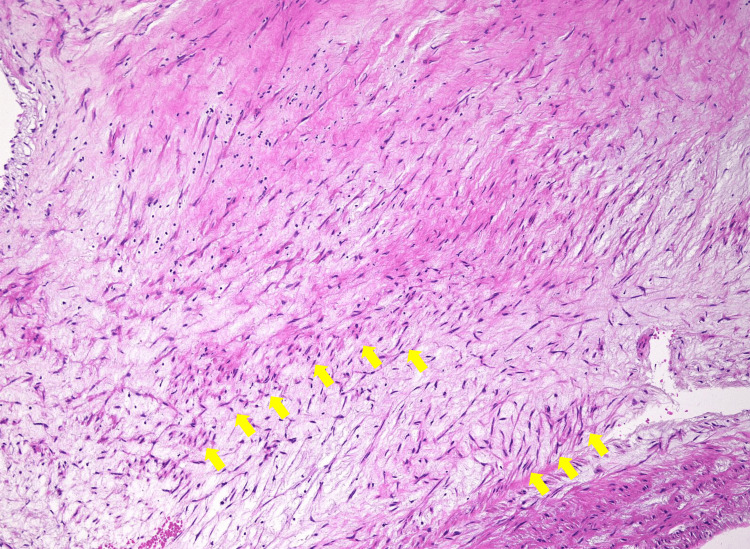
Hematoxylin and eosin staining of the intima of the left internal carotid artery revealed intimal thickening due to fibroelastosis and mucoid degeneration. Hematoxylin and eosin staining revealed prominent myxoid changes extending from the intima to the media, accompanied by the proliferation of immature fibroblasts (yellow arrow).

Given the low NIHSS score and the amount of time elapsed (four hours post onset), the neurosurgeon opted for recanalization therapy. This decision was based on the relatively small lesion observed in imaging compared with the affected vascular territory, along with the consideration that impaired language comprehension would constitute a significant functional disability in light of my professional demands. I received intravenous tissue plasminogen activator therapy along with mechanical thrombectomy. I fell asleep afterward and awoke in the intensive care unit, at which point the nausea and malaise had resolved. For the following week, I received neuroprotective agents, along with dual antiplatelet therapy. Further investigation into the cause of the stroke included tests for coagulation and fibrinolysis disorders, autoimmune diseases, lipid abnormalities, and cardiovascular anomalies, none of which revealed any abnormalities. A CaW was identified on the left carotid artery during CT imaging performed upon admission (Figure 2), and the diagnosis was later confirmed through pathological analysis. Histopathological examination revealed prominent myxoid changes in the intima with increased immature fibroblast proliferation, while atherosclerotic changes were consistent with my age. The media retained its elastic fibers and smooth muscle components, supporting the diagnosis of a CaW (Figure 3).

**Figure 4:**
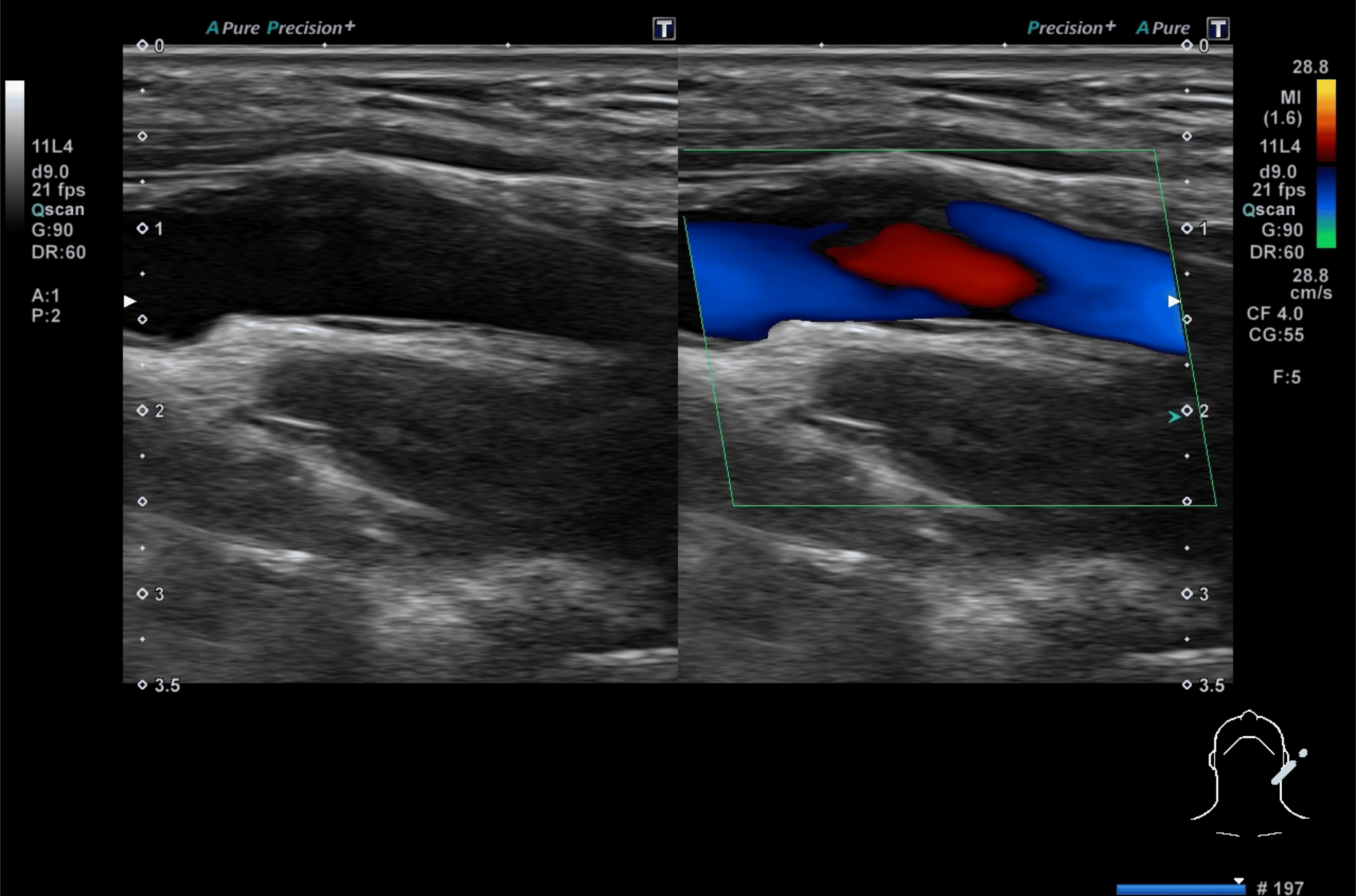
Ultrasound (US) evaluation of the internal carotid artery using conventional and color Doppler techniques. The US assessment of the carotid web (CaW) demonstrated a whirlpool pattern at the level of the CaW in the longitudinal section, observed through conventional imaging and color Doppler flow analysis.

The initial ultrasound, which failed to identify the CaW, underscores the limitations of operator-dependent techniques in detecting subtle arterial abnormalities exclusively in the left carotid artery (Figure 4).

**Figure 5:**
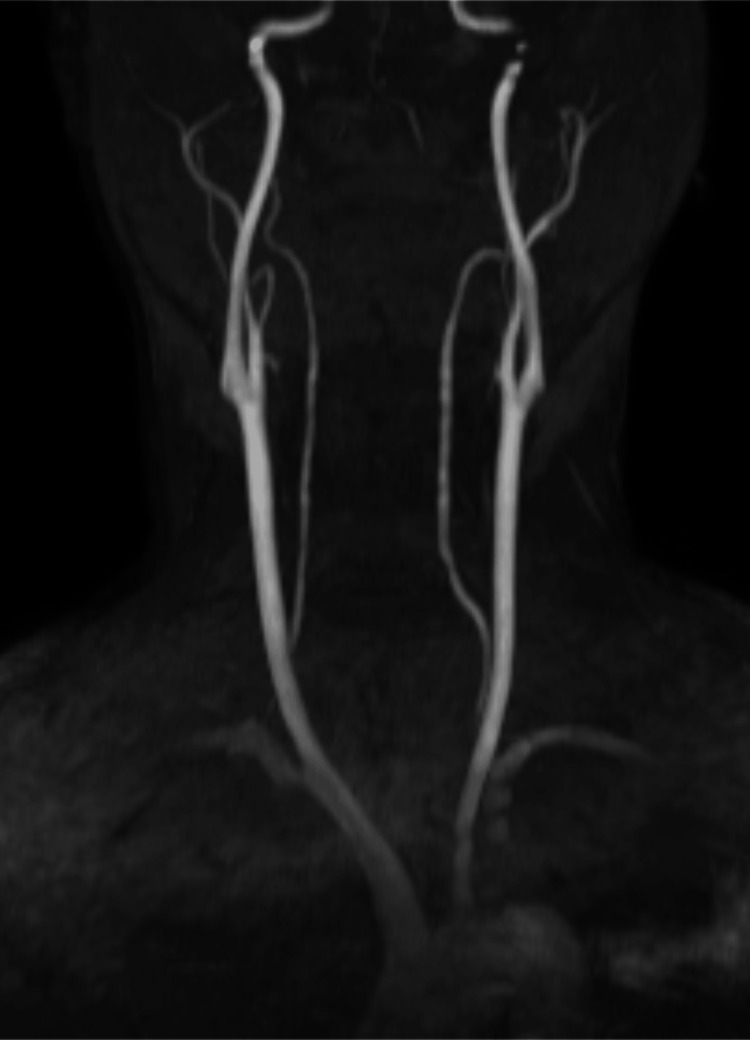
Magnetic resonance angiography (MRA) imaging of bilateral cervical arteries using a time-of-flight (TOF) sequence without contrast. Webs could not be identified in the bilateral carotid arteries.

The success of contrast-enhanced CT in diagnosing CaW highlights the need for more sensitive and standardized imaging protocols for cryptogenic strokes. The CaW could not be identified using magnetic resonance angiography (MRA) (Figure 5).

**Figure 6:**
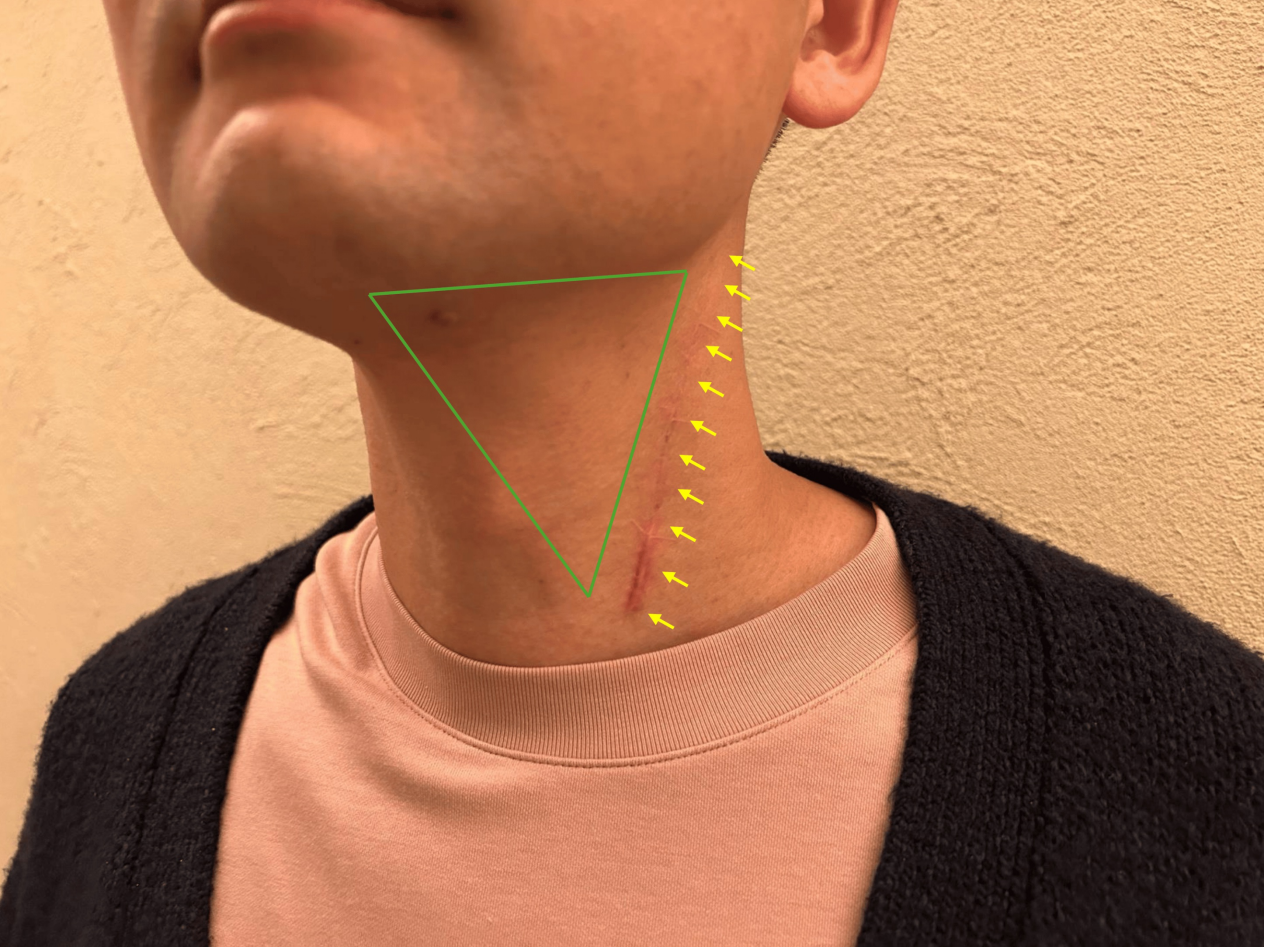
Postoperative photograph of the left cervical region This photograph has been taken six months postoperatively. The surgical site is indicated by a yellow arrow, while the region outlined by a green triangle suffers residual numbness.

There was no physical paralysis; however, aphasia remained as a residual symptom. Initially, I engaged in daily reading of children's picture books and practiced transcription exercises. Gradually, I increased the volume of text, and within approximately two weeks post onset, I was able to read newspapers. Nevertheless, constructing written sentences presented considerable difficulty, requiring around two hours to produce even a four-line paragraph. With continued effort, these skills gradually improved. My hospitalization lasted approximately 45 days. Due to cognitive impairments associated with higher brain dysfunction, I experienced hesitation in independently making decisions and acting. When watching videos, I experienced difficulty processing the information, which may have led to a sensation of head heaviness, ultimately preventing me from continuing to watch. However, through consistent rehabilitation efforts, I have gradually regained self-confidence. One month after being discharged from the hospital, I was able to drive a car with my wife accompanying me. Three months later, I was able to stay overnight alone for the purpose of attending an academic meeting. At present, I have resumed my duties as a pediatrician, including on-call responsibilities, and I experience no significant limitations in daily life. Slight numbness from the surgical site on the right side of my neck to the jaw persists as a minor residual symptom, with the primary inconvenience occurring during shaving (Figure 6).

In addition, mild aphasia persists, which occasionally causes difficulty with word retrieval or articulation when speaking quickly, even when the intended message is clearly conceptualized. This case highlights the importance of recognizing subtle neurological symptoms, such as speech disturbances without paralysis or headache, in otherwise healthy patients.

## Discussion

Here, I reported my experience of acute ischemic stroke caused by a CaW. A CaW is a rare abnormality in the carotid artery where a thin, fibrous flap protrudes into the artery, potentially disrupting blood flow and increasing the risk of blood clots. This condition is increasingly recognized as a cause of otherwise unexplained strokes in young adults. Recent studies suggest that CaWs are prevalent in cases of stroke with otherwise unknown etiologies, particularly among young individuals without other cardiovascular comorbidities. Frequently associated with strokes in the anterior cerebral circulation of unknown cause, CaWs account for approximately 0.27% of all strokes and transient ischemic attacks [5-7].

Although CaWs are rare, accounting for this low frequency of all strokes, they may be underdiagnosed due to the challenges associated with non-invasive imaging. Studies suggest their prevalence may be higher among young patients with cryptogenic strokes, warranting more targeted investigation in this population. Histologically, carotid webs are characterized by focal intimal hyperplasia and medial thickening resulting from hyperplasia of fibrous tissue and smooth muscle [5,8]. Histopathological findings revealed myxoid intimal changes and fibroblast proliferation, consistent with carotid web pathology. These structural abnormalities likely contributed to localized blood flow disturbances, reinforcing the hypothesis that carotid webs act as a nidus for thrombosis and subsequent ischemic events. Carotid web-like cases with intimal thickening have been reported, affecting systemic arteries. These cases exhibit medial hyperplasia in various vascular regions, including the renal arteries, coronary arteries, and intestinal arteries [9-11].

The exact mechanisms underlying the development of a CaW remain unclear, but several hypotheses have been proposed. Considering its typical location on the posterior wall of the proximal internal carotid artery, its development may be explained as follows: First, the carotid artery is formed through the processes of fusion and regression of the third aortic arch and the dorsal aorta. Abnormalities during these developmental stages may contribute to the formation of the carotid web. Second, an imbalance in the development of the arterial wall layers, particularly between the intima and media, during carotid artery formation may play a significant role. Lastly, embryonic blood flow patterns, which are known to greatly influence vascular morphology, may be a factor. It is plausible that abnormal hemodynamic disturbances during development could promote intimal hyperplasia, ultimately resulting in the formation of the CaW [12-14].

**Figure 7:**
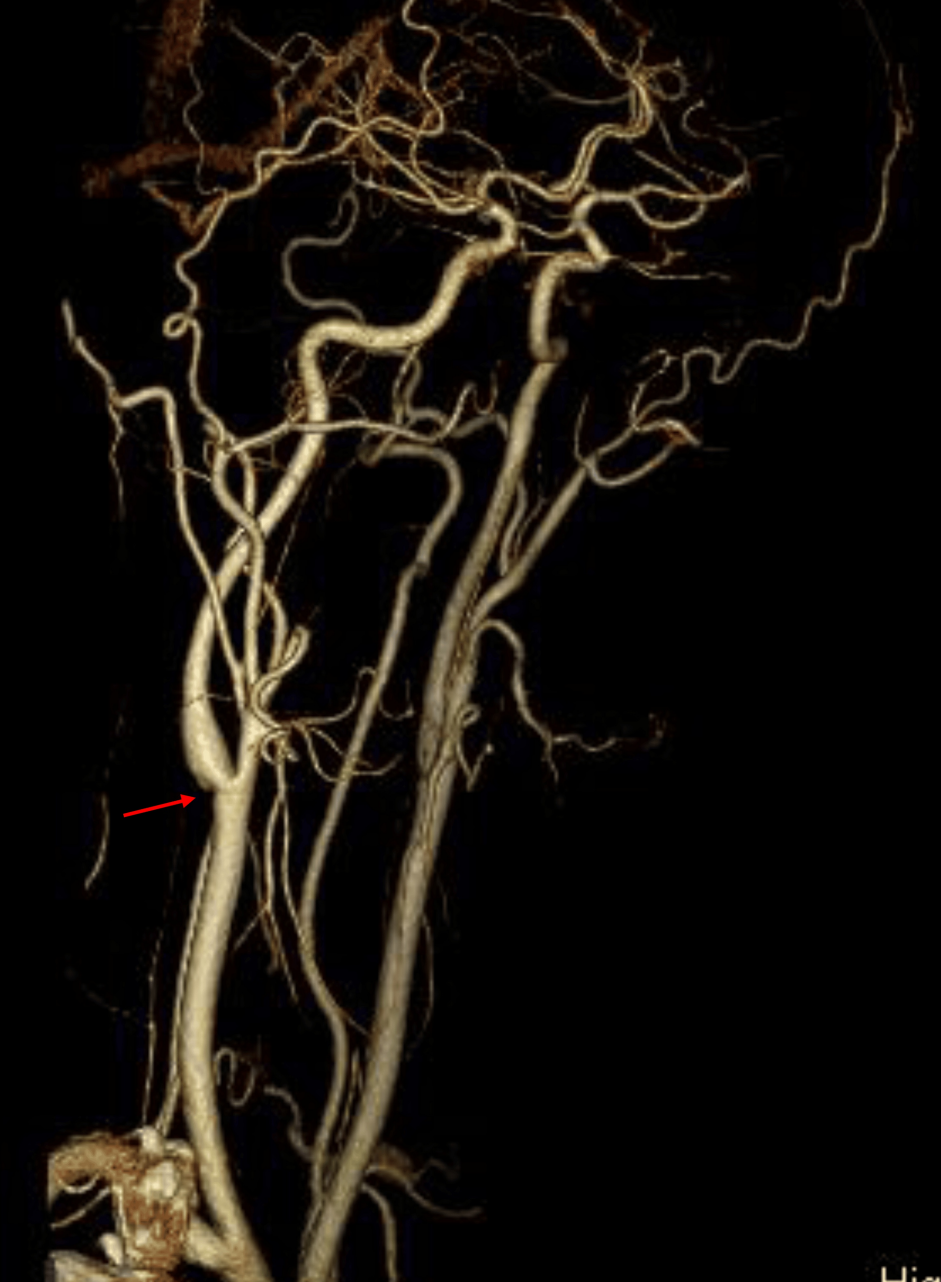
Preoperative sagittal view on computed tomography angiography (CTA) A small web was observed in the right internal carotid artery (red arrow).

The definitive diagnosis of a CaW is established through digital subtraction angiography (DSA); however, in clinical practice, computed tomography angiography (CTA) is the most widely utilized modality. Despite its widespread use, CTA involves radiation exposure and contrast agent administration, making it less suitable for pre-stroke evaluation. Other imaging modalities for assessing CaWs remain under evaluation, with current evidence limited. US is one of the simplest and most accessible imaging modalities. It frequently yields non-specific findings, such as mild bulbar-shaped protrusions, which may be misinterpreted as fibrous plaques of atherosclerotic origin. Comparative studies have demonstrated that US has lower diagnostic accuracy than CTA and conventional DSA. However, experienced sonographers who are well-trained in and familiar with CaW abnormalities may be able to detect these carotid lesions more accurately and with greater ease [7]. Carotid webs can be identified on carotid Doppler US and are optimally visualized in the longitudinal view using B-mode US. A previous study reported that B-mode imaging successfully depicted the characteristic appearance of CaWs in 79% of patients when viewed longitudinally compared with only 38% of cases when viewed in the axial plane [15]. The likely cause of the initial oversight was related to the operator’s technique because this type of structure can be missed if the probe does not make proper contact. Specifically, when the probe is positioned perpendicularly (at a 90° angle) to the lesion, sound waves are more likely to reflect directly back, producing stronger echoes and a clearer image. However, if the angle deviates from this perpendicular orientation, the reflected waves weaken, potentially making certain areas difficult to visualize or resulting in a blurred image [16]. As an alternative approach, contrast-enhanced US, which is likely to better identify and assess arterial wall irregularities and plaque vulnerability in carotid atherosclerosis, might enhance visualization of the slit-like protrusion of a CaW, which is not detected with conventional US. This method lacks simplicity and has not undergone a validation study [7]. In my own case, the initial US examination failed to identify the CaW. The missed diagnosis during the initial ultrasound examination highlights the potential for false negatives in cases where subtle vascular abnormalities are present. This underscores the importance of operator training and the possible role of advanced techniques, such as contrast-enhanced ultrasound or 3D imaging, in improving diagnostic accuracy. By contrast, a CT scan, with its 360° CT beam coverage, did not miss the CaW. In fact, a smaller CaW was observed on the right side compared with the left side by CTA on admission (Figure 7), but a detailed examination using US during both evaluations failed to identify the small CaW on my right carotid artery.

While US is widely accessible, its sensitivity for detecting CaWs depends heavily on operator expertise and imaging technique. By contrast, CTA offers higher sensitivity and 360-degree visualization, making it a more reliable diagnostic tool. However, the associated risks of radiation and contrast exposure limit its routine use for pre-stroke evaluation.

Brain MRI scans are highly effective for high-resolution visualization of brain structures, facilitating the early detection of conditions such as brain tumors, cerebral infarctions, cerebral hemorrhages, Alzheimer’s disease, and dementia. Furthermore, MRA is particularly valuable for identifying small vessel abnormalities in the brain, aiding the early assessment of stroke and dementia risk [17]. An MRA can be acquired using a time-of-flight (TOF) sequence without the need for contrast agents, leveraging the enhancement associated with blood flow. Notably, TOF sequences can be performed with two-dimensional (2D) acquisitions. However, a limitation of 2D imaging is its reduced sensitivity to in-plane flow, which is particularly significant when assessing structures such as CaWs [18]. This limitation arises because MRA is not well-suited for identifying blood flow patterns around such structures. The diagnostic accuracy of MRA in detecting CaWs remains limited. Although MRA can visualize CaWs and offers the advantage of detailing wall morphology and composition, the routine TOF protocol with 2D acquisition is less sensitive to slow or retrograde flow. Consequently, its ability to effectively delineate CaWs is constrained [7]. For example, my CaW could not be identified using routine MRA techniques. (Figure 4) When a specialized sequence is used, the detection of CaWs can be highly reliable. For example, the advanced techniques of three-dimensional contrast MRA and multi-contrast fast spine echo imaging can yield higher diagnostic accuracy [7]. However, such sequences are typically ordered only in cases where a CaW is suspected, meaning it may be overlooked in routine MRA examinations. Further discussion is warranted to determine whether the implementation of specialized MRI sequences should be standard practice. Prospective treatment strategies for my condition will be documented here.

As noted above, a small CaW is present in the right carotid artery, and mild neurological sequelae remain. General observational data on CaWs indicate that, despite medical management, patients with symptomatic CaWs may face up to a 20% risk of stroke recurrence within two years. While both stenting and carotid endarterectomy are considered effective treatments for CaWs, the choice between these modalities remains a subject of ongoing debate. Given the potential complication of bilateral recurrent laryngeal nerve paralysis associated with bilateral carotid surgeries, stenting is planned for the intervention on the right side [19]. 

Given the risk of recurrence associated with CaWs and the limitations of non-invasive diagnostic methods, further studies are needed to determine the optimal timing for surgical intervention. The choice between stenting and carotid endarterectomy, as well as the role of conservative management, should be informed by better diagnostic accuracy and a clearer understanding of the pathophysiology of CaWs. This could lead to more personalized treatment strategies and improved long-term outcomes. Residual symptoms, such as mild aphasia and numbness, are consistent with infarction in the left middle cerebral artery territory. These findings reflect the importance of intensive rehabilitation in mitigating long-term deficits, particularly in patients with high professional or cognitive demands. Given the diagnostic challenges associated with carotid webs, clinicians should consider a multimodal imaging approach in cases of cryptogenic stroke, particularly in younger patients. Advanced imaging techniques, such as contrast-enhanced CT or specialized MRA sequences, may be warranted when ultrasound findings are inconclusive. Incorporating artificial intelligence (AI) into imaging analysis and developing standardized diagnostic protocols will also be crucial for improving early detection and risk stratification of carotid artery lesions [20]. Specifically, further investigations on the size and morphologies of CaWs and their associations with stroke recurrence risk might be needed to optimize treatment strategies personalized for each patient [5].

## Conclusions

I, a 46-year-old male with no significant risk factors, presented with an acute ischemic stroke, which was subsequently determined to be caused by a CaW. While DSA and CTA are effective in confirming CaWs, they are invasive. While invasive imaging modalities such as DSA can confirm the presence of CaWs in patients who have experienced a stroke, their routine use for screening in asymptomatic patients remains uncertain. The limitations of non-invasive techniques underscore the need for better diagnostic criteria and possibly broader screening practices in younger patients at risk of cryptogenic stroke. As a specific modality, US and MRA can occasionally identify CaWs when performed by experienced operators or with specialized imaging techniques. Nevertheless, accurate detection of CaWs in routine clinical practice using these non-invasive methods remains a significant challenge. The role of contrast-enhanced US and 3D MRA should be explored further, not only for better detection rates but also to refine patient selection for surgical or endovascular interventions. This approach could improve outcomes in the management of cryptogenic stroke due to CaWs. The pathogenesis of CaWs remains uncertain, and our case highlights the diagnostic challenges, particularly the limitations of US and MRA in detecting these lesions. The need for more sensitive diagnostic techniques, such as contrast-enhanced imaging, is evident from the failure to identify the CaW in this case. This underscores the importance of further research to clarify the development of CaWs and to improve diagnostic protocols.
